# The Use of Proteomic Tools to Address Challenges Faced in Clonal Propagation of Tropical Crops through Somatic Embryogenesis

**DOI:** 10.3390/proteomes6020021

**Published:** 2018-05-04

**Authors:** Chiew Foan Chin, Hooi Sin Tan

**Affiliations:** School of Biosciences, The University of Nottingham Malaysia Campus, Jalan Broga, 43500 Semenyih, Selangor Darul Ehsan, Malaysia; hooisin_86@hotmail.com

**Keywords:** tissue culture, tropical crops, proteomics, somatic embryogenesis

## Abstract

In many tropical countries with agriculture as the mainstay of the economy, tropical crops are commonly cultivated at the plantation scale. The successful establishment of crop plantations depends on the availability of a large quantity of elite seedling plants. Many plantation companies establish plant tissue culture laboratories to supply planting materials for their plantations and one of the most common applications of plant tissue culture is the mass propagation of true-to-type elite seedlings. However, problems encountered in tissue culture technology prevent its applications being widely adopted. Proteomics can be a powerful tool for use in the analysis of cultures, and to understand the biological processes that takes place at the cellular and molecular levels in order to address these problems. This mini review presents the tissue culture technologies commonly used in the propagation of tropical crops. It provides an outline of some the genes and proteins isolated that are associated with somatic embryogenesis and the use of proteomic technology in analysing tissue culture samples and processes in tropical crops.

## 1. Introduction

It is anticipated that, by 2050, the population in the tropics will reach 50% of the world’s population [1]. This population increase will pose a threat to the available resources, including food for consumption. Tropical crop production, therefore, needs to be increased to cater to the burgeoning demand. This can be accomplished with effective crop improvement strategies. Plant tissue culture is one such crop improvement strategy, which has been widely adopted not only for mass propagation of elite planting materials, but to capture maximum yield potential for field planting.

Plant tissue culture is an important tool in plant biology that has been widely used for basic and applied research. Most plant cells display developmental plasticity, i.e., with the capability to dedifferentiate, redifferentiate, and regenerate into a whole plant, known as totipotency. Plant researchers recognise this attribute to be a useful feature in using plants as a model organism for studying biological, biochemical, physiology, and molecular mechanisms in living systems [2]. More recently, Sugimoto et al. [3] suggested that the totipotency of plant cells might not be applicable to all plant cells, but restricted only to a group of stem cells. These plant stem cells are widely found in plants due to their presence in the vascular systems, specifically the pericycle region. This could account for why regeneration in plant tissue culture is more amenable in dicotylenous compare to monocotyledous plants.

There are many advantages associated with plant tissue culture compared with conventional plant propagation [4]. In plant tissue culture, optimal growth and physical conditions are provided in order to maximise growth parameters of the plants for divisions and multiplications. Therefore, through in vitro clonal micropropagation, the maximum yield potential of the plant can be captured by mass multiplication of selected elite plants as planting materials. This strategy has been practiced in the production of many tropical field crops, including oil palm [5], banana [6], pineapple [7], rubber [8], and tropical fruit trees [9].

Even though commercial plant tissue culture laboratories have been established to supply elite field planting materials for many years, there are hurdles that needed to be overcome in order to implement the technology successfully. Some of the main challenges facing mass micropropagation are genotype-dependant, somaclonal variations and the low conversion rate of tissue culture materials from explants to plantlets. As a result, large economic losses have been incurred in the form of labour, resources, and time.

Several molecular tools have been used in an attempt to unravel the biological and molecular mechanisms underlying the regeneration competency in plant tissue culture samples [10]. The emergence of proteomic technology provides us with a powerful tool to investigate the proteins, which are the gene products that are directly associated with the phenotypic traits under study. In this mini review, we provide a glimpse of the use of proteomic technologies to address challenges faced in the tissue culture of tropical crops.

## 2. Tissue Culture in Tropical Crops

With the onset of modern agriculture, many tropical crops with lucrative potential, such as oil palm, rubber, coffee, banana, pepper, cocoa, and pineapple, were planted at the large-scale in the field. This has resulted in a significant demand for high-quality planting materials in order to boost the yield at harvest. Planting materials supplied through the route of tissue culture serve as a viable alternative for the establishment of tropical crop plantations. Therefore, many commercial plant tissue culture laboratories have been established.

Plant tissue culture for the mass production of planting materials can be carried out in several ways. Explant materials from various plant parts, including leaf, stem, root, shoot, meristem, hypocotyl, cotyledon, embryo, pollen, and flower, can be used as starting materials for clonal multiplication under in vitro aseptic conditions [10]. Normally, differentiated somatic cells are involved, hence the term somatic embryogenesis. Micropropagation by somatic embryogenesis can be accomplished through direct or indirect routes. The direct route involves the growth of adventitious shoots or roots directly from the differentiated tissues. Usually, only a few clonal plants can be obtained through the direct method. Therefore, the direct method is not commonly adopted for commercial mass propagation even though this method has been known to produce plants that are genetically more stable. Somatic embryogenesis via the indirect route is the generally preferred tissue culture process for commercial tissue culture laboratories as this method has significant potential to produce hundreds of thousands of somatic embryos with the use of a small number of starting explant materials.

The indirect route of somatic embryogenesis usually involves an initial dedifferentiation of explants into the callus phase. The callus may or may not develop further into embryos depending on the embryogenic competence acquired by the callus [11]. The induction of callus and its subsequent conversion into embryos has been linked with endogenous and exogenous auxin. The application of the auxin analogue, 2,4-dichlorophenoxyacetic acid (2,4-D), has been known to trigger plant cells to proliferate into undifferentiated cells [12], however, the conversion of the callus into embryos requires it to be grown in hormone free medium. Callus induction was reported to have involved the termination of the current gene expression in the explant tissue, which was then replaced by an embryogenic gene expression programme [13]. The current gene expression may be downregulated by DNA methylation, influenced by the plant hormone auxins [14], which would explain the role of auxin in the process of somatic embryogenesis.

The understanding of the developmental trajectory of somatic embryos from explants will help us address some of the challenges faced by commercial tissue culture laboratories for the large-scale production of elite planting materials. To this end, many researchers have attempted to use genome information to isolate gene sequences associated with plant embryogenesis.

## 3. Regulatory Genes in Somatic Embryogenesis

**Figure 1:**
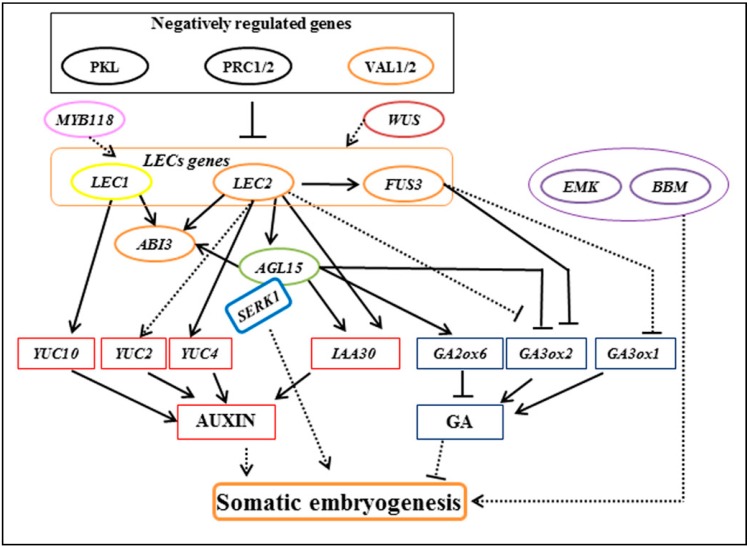
Regulatory genes controlling somatic embryogenesis in *Arabidopsis* (adapted with permission from Guan et al. (2016) [15]).

Several genes involved in regulating somatic embryogenesis have been isolated. Most of the work has been carried out using the model plant species, *Arabidopsis thaliana* (Figure 1—[15]). Although the genome sequence of *Arabidopsis* differs from crop genomes, the gene catalogue provides a valuable resource for orthogonal gene comparison and studies with other crop species. Studies found that *LEAFY COTYLEDON1* (*LEC1*) and *LEC2* are genes involved with one of the key pathways in somatic embryogenesis [16]. Overexpression of LEC genes trigger the upregulation of YUCCA (YUC) genes, which, in turn, leads to an increase in the endogenous levels of auxin in *Arabidopsis* [17]. Using auxin polar transport inhibitors, Liu et al. [18] has shown that auxin is involved in embryo morphogenesis. Other genes involved in somatic embryogenesis are *SOMATIC EMBRYOGENENSIS RECEPTOR KINASE* (*SERK*) [19], *BABY BOOM* (*BBM*) [20], and *WUSCHEL-RELATED HOMEOBOX* (*WOX*) [21]. The use of information at the genome level has allowed the elucidation of certain functions in the cells. However, several studies revealed that the levels of transcripts and proteins often do not correlate well [22]. Therefore, the use of proteomic technology to elucidate cellular and molecular mechanisms underlying important processes, such as somatic embryogenesis, is essential.

## 4. The Development of Proteomic Technology in Crops

The word “proteome” is derived from PROTEins expressed by a genOME. Proteomics is the characterization of the entire protein complement expressed by a genome of a given organism [23]. The proteome is the time- and cell-specific protein complement of genome in a cell at any given time. It is the large-scale study of protein properties, such as expression, modification, and interaction, to gain an overview of cellular processes at the protein level.

Since proteins are the gene products of the cells, they provide a better representation of the linkage to the phenotypic traits of the organism. Proteomics reveals cellular functions at the level of the cell, organ, tissue, and organism, which enables a more in-depth study of molecular and biological processes to be accomplished.

The most intensive studies of proteomics have been performed on the model plant species *Arabidopsis thaliana* and rice, particularly after the publication of the genome sequence drafts of *Arabidopsis* [24] and rice [25,26] in 2000 and 2002, respectively. This is because genomic information facilitates protein identification. Similarly, with increasing genomic DNA and EST sequencing data deposited into public domain databases, increasing the number of crops studied using proteomic approaches, for example, maize [27], wheat [28], barley [29,30], soy bean [31], chickpea [32], and date palm [33].

With the advancements of proteomic approaches, there are many dimensions of studies that can be accomplished by using the techniques that have been developed. In general, proteomics can be subdivided into different areas, including descriptive proteomics, differential expression proteomics, posttranslational modification, interactomics, and proteinomics [34]. The proteomic techniques can be further categorized into gel-based and gel-free proteomics.

The most common gel-based technique used in a proteomic laboratory is two-dimensional gel electrophoresis (2-DE) developed in the mid-1970s [35]. This technique is popular because it is relatively easy and inexpensive to set up. 2-DE approaches have been reported to be one of the most powerful tools for protein profiling to visualize isoforms that results from charged posttranslational modifications [36]. However, conventional 2-DE techniques have the limitation of gel-to-gel variations which may introduce technical errors to the results. Differential gel electrophoresis (DIGE) technology developed in the early 2000s [37,38,39] has helped to mitigate this shortcoming. DIGE is normally used for comparative proteomic studies, although it has protein co-migration problems. More recently, the development of 3-D gel separation is reported to have helped to reduce this co-migration issue [40].

In addition, 2DE has other limitations such as quantitative reproducibility, poor detection of certain proteins, including low-abundance proteins, acidic or basic proteins, hydrophobic proteins, or proteins with extreme sizes. The development of gel-free proteomics, therefore, provides an alternative to more accurately quantitate protein and enable deeper explorations of complex proteins. One of the main attractions of the gel-free proteomic approach is the number of proteins that can be identified. It has been reported that over 12,000 proteins have been identified in different organs of *Arabidopsis* [41] using gel-free proteomic approaches, compared with 4000 identified proteins in rice using gel-based approaches [42]. The high number of identified proteins in the gel-free approach implies a wider coverage of proteins being identified which could include the low-abundance proteins. Therefore, more recent proteomic studies in crops, such as wheat [43,44] and soy beans [45,46], have adopted gel-free proteomic approaches.

Both the gel-based and gel-free approaches use mass spectrometry for protein identification. Proteomic technologies have made a big leap with the discovery of protein ionisation methods, notably the electrospray ionisation (ESI) and matrix-assisted laser desorption/ionization (MALDI) techniques in mass spectrometry, which enable proteins to be identified. Mass spectrometry (MS)-based proteomics can be used for protein profiling, protein identification, and quantification, as well as analysis of protein modifications and interactions [47].

## 5. Proteomics in Somatic Embryogenesis

The current progress in the field of proteomics provides a concrete platform to study the molecular changes occurring in somatic embryogenesis in plants. 2DE has been applied in somatic embryogenesis studies in carrot [48], cichorium [49], Vitis [50], *Cupressus sempervirens* [51], and *Cyclamen persicum* Mill [52].

Three major somatic embryogenesis-related proteins have been identified in cichorium as pathogenesis-related (PR) proteins [49]. Using embryogenic and non-embryogenic cichorium ‘474’ cell lines, the study showed that an increase in protein level of up to eight-fold was observed in the embryogenic cells compared to the non-embryogenic cells. The proteins associated with somatic embryogenesis were identified to be β-1,3-glucanase, chitinase, and osmotin-like proteins. Since all three proteins are known to be related to stress, it implies that these proteins could perform multiple roles in the cells, including cell development.

In a study on *Picea glauca*, a total of 48 differentially-expressed proteins were identified across four stages of somatic embryo development [53]. The differential protein abundance could be detected as early as seven days post embryo development using 2DE coupled with MS/MS proteomic technology. The most abundant protein was found to be the storage protein vicilin, which has similarly been found to be the most abundant polypeptide found in zygotic protein [54]. The other significant protein found in association with somatic embryo development is enolase, which has shown to be induced during anaerobiosis in maize [55].

Proteomic analysis has been used to study the induction of somatic embryos in *Medicago truncatula* culture grown in P4 media supplemented with 6-Benzylaminopurine and 1-Naphthaleneacetic acid [56]. A total of 54 differentially-abundant proteins were found, however, only 16 proteins were identified. The results showed that Rubisco small chain proteins gradually decreased over the growth period of the cultured tissue, suggesting their possible role as markers for tissue differentiation and proliferation. Other important proteins identified were Thioredoxin H protein, which is associated with early development of somatic embryogenesis and 1-Cys Peroxiredoxin, which plays a role in late embryogenesis. In another similar study by Jong et al. [57] on *Medicago truncatula* protoplast proliferations, a total of 886 protein spots with differential abundance were detected, out of which, 89 proteins were identified. The majority of the proteins were categorized under the main cellular processes such as energy metabolism, defence, or stress responses, secondary metabolism and protein synthesis, suggesting that protoplast proliferation involves cellular reorganizations.

A proteomic analysis was carried out on various developmental stages of somatic embryos in *Cyclamen persicum* Mill by Bian et al. [52]. The study found 35 differential protein spots with 10 protein spots identified. Examples of the identified proteins are the proteasome subunit and triosephosphate isomerase. The proteasome subunit has reported to be closely related to cell proliferation processes [58] and is involved in early somatic embryo development in *Picea glauca* [53]. Triosephosphate isomerase was found to be involved in sugar metabolism and is one of the key regulatory enzymes involved in glycolysis and the tricarboxylic acid cycle [59].

With the development of gel-free proteomic technology, more proteins associated with somatic embryogenesis have been detected and identified. An extensive review on the use of gel-free proteomics to study somatic embryogenesis has been provided by Heringer et al. [60]. The somatic embryogenesis-related proteins were classified into different groups. For example, stress and detoxification is one of the groups that is associated with somatic embryos development because the developmental process involves genetic reprogramming, cell dedifferentiation, and maintenance of cellular homeostasis.

## 6. Application of Proteomics in Tissue Culture of Tropical Crops

The incorporation of high-throughput “-omics” technologies, principally genomics, transcriptomics, proteomics, and metabolomics, has facilitated the discovery pathways for the functionality of genes in a systematic manner. Proteomics provides a platform towards understanding cellular functions at the level of cells, organs, tissues, and organisms. Proteomics is a promising approach that can complement and relate to transcriptomics and metabolomics [61]. This approach has become one of the main technologies chosen by plant researchers to unravel the fundamental and molecular levels of cells in order to characterize plant subspecies, and to identify putative molecular markers to aid in crop breeding programs [62].

The most intensive proteomic studies have been conducted using the model plant species *Arabidopsis thaliana* and rice, which is reflected in the highest number of publications found in proteomic related references. Publications of plant proteome research increased steeply after the completion of the genome sequence of *Arabidopsis* [24] and rice [25]. Today, many plant biologists have adopted proteomic technology in their research studies, which have extended to non-model plant species, such as food crops [62].

Proteomics can be used to investigate many biological processes in crops. Since tissue culture technology plays an important role in plant breeding programmes, the technology has been widely adopted by plantation research companies. However, there are still hurdles which impede the progress of tissue culture technology. For example, some of the problems in dire need of attention include the genotypically-dependent nature of the culture, a low conversion rate of embryogenic competent tissue, and heterogeneity of the culture samples. Proteomic technology serves as a promising tool to unravel biological processes and molecular mechanisms in the plants down to the cellular level. This will help researchers to better predict the outcomes should any biological problems arise. The use of proteomic tools, therefore, have been adopted in the tissue culture of tropical crops studies (Table 1). Nevertheless, most of the studies focussed on somatic embryogenesis for use in mass propagation of clonal planting materials due to its potential in meeting the industrial requirements.

Most proteomics studies have involved the initial conversion of explants to proembrogenic callus or embrogenic callus in somatic embryogenesis. This is because the initial phase is the critical stage to screen for non-embrogenic calli to be discarded so that resources, such as labour and culture media, can be saved. It is interesting to note that the proteins involved in the induction of embryogenic calli at the initial stage of somatic embryogenesis for most of the tropical crop species studied have been associated with glycolysis, stress, or defence mechanisms (Table 2). Starch is essential for initial embryo development and it is synthesised through glycolysis. The exhibition of oxidative stress in in vitro culture has been documented [75] and plants generally have defence mechanisms against reactive oxygen species (ROS) in order to overcome oxidative stress during the culturing process.

## 7. Conclusions

Proteomics serve as a promising platform for researchers to study total proteins at the global cell level. The information derived from proteomics will help us to uncover many complex questions underlying biological processes in the cell. Since plant tissue culture involves the manipulation of cells or tissues under in vitro conditions, proteomics serves as an effective tool to be used in addressing the questions of cell growth and division. As can be seen in the elucidation of some protein species associated with the initial formation of embryogenic calli, such information would enable us to make more accurate predictions about the fate of the cells undergoing tissue culture.

Despite great promise being brought about by proteomic technology, the technique is not widely adopted in the improvement programs of tropical crops. This could be due to the dynamic range of proteins that make it difficult to be used as an effective application tool, for example, as potential biomarkers. In addition, protein is an unstable macromolecule which is, therefore, not practical to handle in bulk.

Nevertheless, as the field of proteomic technologies advances, the sensitivity of protein detection will be enhanced. This has been seen in the use of gel free proteomic approaches. Even though the use of such an approach is currently not widely adopted due to the prohibiting cost of setting up the facilities and the high operating cost, it is hoped that, over time, the cost will eventually come down to allow more researchers to gain access to the technology. We believe that this would open up new avenues for us to solve more complex challenges encountered in tissue culture.

## Figures and Tables

**Table 1:** Applications of proteomic technology in tissue culture of tropical crops.

Scientific Name (Common Name)	Explants	Micropropagation Methods	Proteomic Techniques	References
*Coffea arabica* (coffee)	Leaf	SE	Q Exactive Orbitrap MS	[63]
*Cyphomandra betacea* (tamarillo)	Leaf	SE	LC MS/MS	[64]
*Elaeis guineensis* (oil palm)	(a) Leaf (b) Zygotic embryos	SE	2-DE & MALDI ToF MS/MS	[65,66]
*Manihot esculenta* (cassava)	Green cotyledons of somatic Embryos	SE	2-DE/MALDI-MSMS	[67]
*Musa spp.* (banana)	Immature male flower buds	SE	2-DE & MALDI-Tof MS	[68]
*Persea americana* (avocado)	Immature zygotic embryos	SE	2D-DIGE	[69]
*Phoenix dactylifera* (date palm)	Embryos	SE	2-DE & MALDI-Tof MS/MS	[70]
*Saccharum spp.* (sugarcane)	nodal segments with axillary buds	SE	ESI-QTOF HDMS	[71]
*Theobroma cacao* (cocoa)	(a) Flower, zygotic embryos (b) Zygotic embryos	SE	(a) 2DE and nano-LC-MS (b) 2DE and EASY-nLC coupled with Micro-ToF-Q	[72,73]
*Vanilla planifolia* (vanilla)	nodal segments with axillary buds	OG	2DE and MALDI Tof/Tof/MS	[74]

**Table 2:** Proteins involved in the early embryogenic callus formation in somatic embryogenesis.

Plant Type	Protein	Cellular Functions	References
*Cyphomandra betacea* (tamarillo)	fructokinase	glycolysis	[64]
	Pathogenesis-related proteins	Stress association	
	Heat shock 70 kDa	Stress association	
	enolase	glycolysis	
*Elaeis guineensis* (oil palm)	triosephosphate isomerase	glycolysis	[65]
	L- ascorbate peroxidase	Defence response	
	superoxide dismutase	defence response	
*Elaeis guineensis* (oil palm)	type IIIa membrane protein cp-wap13	cell wall degradation, loosening and biosynthesis	[66]
	fructokinase	glycolysis	
	PR proteins (peroxidase and glutathione S-transferase)	Stress association	
*Musa spp.* (banana)	indole-3-pyruvate monooxygenase	Auxin synthesis	[68]
	adenylate isopentenyltransferase	Cytokinin synthesis	
	Acyl-acyl-carrier-protein desaturase	fatty acid biosynthesis	
	pectinesterase inhibitor	Inhibit pectin accumulation	
	Caffeoyl-CoA O-methyltransferase	lignin biosynthesis	
*Persea americana* (avocado)	superoxide dismutase	Defence response	[69]
	Heat shock 70 kDa	Stress association	
	Glutathione S-transferase	Detoxification process	
*Theobroma cacao* (cocoa)	β-1,3 glucanases	Stress association	[72]
	chitinase	Stress association	
	osmotin-like protein	Stress association	

## References

[1] Wilkinson A. (2014). Expanding tropics will play greater global role, report predicts. Science.

[2] Thorpe T.A. (2007). History of Plant Tissue Culture.

[3] Sugimoto K., Gordon S.P., Meyerowitz E.M. (2011). Regeneration in plants and animals: Dedifferentiation, transdifferentiation, or just differentiation?. Trends Cell Biol..

[4] Brown D.C.W., Thorpe T.A. (1995). Crop improvement through tissue culture. World J. Microbiol. Biotechnol..

[5] Staritsky G. (1970). Tissue culture of the oil palm (*Elaeis guineensis* Jacq.) as a tool for its vegetative propagation. Euphytica.

[6] Alvard D., Cote F., Teisson C. (1993). Comparison of methods of liquid medium culture for banana Micropropagation—Effects of temporary immersion of explants. Plant Cell Tissue Organ Cult..

[7] Sripaoraya S., Marchant R., Power J.B., Davey M.R. (2003). Plant regeneration by somatic embryogenesis and organogenesis in commercial pineapple (*Ananas Comosus* L.). In Vitro Cell. Dev. Biol. Plant.

[8] Zhao H., Peng M., Wang X., Zeng H.C., Chen X.T. (2009). Micropropagation of rubber tree (*Hevea brasiliensis*) by employing mature stem as explants. Genom. Appl. Biol..

[9] Gavinlertvatana P. (1992). Commercial micropropagation of tropical fruit trees. Acta Hortic..

[10] Vasil I.K. (1991). Plant tissue culture and molecular biology as tools in understanding plant development and in plant improvement. Curr. Opin. Biotechnol..

[11] Namasivayam P. (2007). Acquisition of embryogenic competence during somatic embryogenesis. Plant Cell Tissue Organ Cult..

[12] Feher A., Pasternak T.P., Dudits D. (2003). Transition of somatic plant cells to an embryogenic state. Plant Cell Tissue Organ Cult..

[13] Von Arnold S., Sabala I., Bozhkov P., Dyachok J., Filonova L. (2002). Developmental pathways of somatic embryogenesis. Plant Cell Tissue Organ Cult..

[14] Loschiavo F., Pitto L., Giuliano G., Torti G., Nuti-Ronchi V., Marazziti D., Vergara R., Orselli S., Terzi M. (1989). DNA methylation of embryogenic carrot cell cultures and its variations as caused by maturation, differentiation, hormones and hypomethylation drugs. Theor. Appl. Genet..

[15] Guan Y., Li S.G., Fan X.F., Su Z.H. (2016). Applications of somatic embryogenesis in woody plants. Front. Plant Sci..

[16] Elhiti M., Stasolla C., Wang A. (2013). Molecular regulation of plant somatic embryogenesis. In Vitro Cell. Dev. Biol. Plant.

[17] Stone S.L., Braybrook S.A., Paula S.L., Kwong L.W., Meuser J., Pelletier J., Hsieh T.F., Fischer R.L., Goldberg R.B., Harada J.J. (2008). Arabidopsis LEAFY COTYLEDON2 induces maturation traits and auxin activity: Implications for somatic embryogenesis. Proc. Natl. Acad. Sci. USA.

[18] Liu C.M., Xu Z.H., Chua N.H. (1993). Auxin polar transport is essential for the establishment of bilateral symmetry during early plant embryogenesis. Plant Cell.

[19] Schmidt E.D., Guzzo F., Toonen M.A., de Vries S.C. (1997). A leucine-rich repeat containing receptor-like kinase marks somatic plant cells competent to form embryos. Development.

[20] Boutilier K., Offringa R., Sharma V.K., Kieft H., Ouellet T., Zhang L., Hattori J., Liu C.M., van Lammeren A.A., Miki B.L. (2002). Ectopic expression of BABY BOOM triggers a conversion from vegetative to embryonic growth. Plant Cell.

[21] Zuo J., Niu Q.W., Frugis G., Chua N.H. (2002). The WUSCHEL gene promotes vegetative-to-embryonic transition in Arabidopsis. Plant J..

[22] Tobias M., Marc G., Luis S. (2009). Correlation of mRNA and protein in complex biological samples. FEBS Lett..

[23] Wilkins M.R., Gasteiger E., Gooley A.A., Appel R.D., Humphery-Smith I., Hochstrasser D.F., William K.L. (1996). Progress with proteome projects: Why all protein expressed by a genome should be identified and how to do it. Biotechnol. Genet. Eng. Rev..

[24] The Arabidopsis Genome Initiative (2000). Analysis of the genome sequence of the flowering plant Arabidopsis thaliana. Nature.

[25] Goff S.A., Ricke D., Lan T.H., Presting G., Wang R., Dunn M., Glazebrook J., Sessions A., Oeller P., Varma H. (2002). A Draft Sequence of the Rice Genome (*Oryza sativa* L. ssp. *japonica*). Science.

[26] Yu J., Hu S., Wang J., Wong G.K.S., Li S., Liu B., Deng Y., Dai L., Zhou Y., Zhang X. (2002). A Draft Sequence of the Rice Genome (*Oryza sativa* L. ssp. *indica*). Science.

[27] Majeran W., Friso G., Ponnala L., Connolly B., Huang M., Reidel E., Zhang C., Asakura Y., Bhuiyan N.H., Sun Q. (2010). Structural and Metabolic Transitions of C4 Leaf Development and Differentiation Defined by Microscopy and Quantitative Proteomics in Maize. Plant Cell.

[28] Peng Z., Wang M., Li F., Lv H., Li C., Xia G. (2009). A proteomic study of the response to salinity and drought stress in an introgression strain of bread wheat. Mol. Cell. Proteom..

[29] Moller A.L.B., Pedas P., Andersen B., Svensson B., Schjoerring J.K., Finnie C. (2011). Responses of barley root and shoot proteomes to long-term nitrogen deficiency, short-term nitrogen starvation and ammonium. Plant Cell Environ..

[30] Rasoulnia A., Bihamta M.R., Peyghambari S.A., Alizadeh H., Rahnama A. (2011). Proteomic response of barley leaves to salinity. Mol. Biol. Rep..

[31] Komatsu S., Makino T., Yasue H. (2013). Proteomic and Biochemical Analyses of the Cotyledon and Root of Flooding-Stressed Soybean Plants. PLoS ONE.

[32] Subba P., Kumar R., Gayali S., Shekhar S., Parveen S., Pandey A., Datta A., Chakraborty S., Chakraborty N. (2013). Characterisation of the nuclear proteome of a dehydration-sensitive cultivar of chickpea and comparative proteomic analysis with a tolerant cultivar. Proteomics.

[33] Marondedze C., Gehring C., Thomas L. (2014). Dynamic changes in the date palm fruit proteome during development and ripening. Hortic. Res..

[34] Jorrín-Novo J.V., Pascual J., Sánchez-Lucas R., Romero-Rodríguez M.C., Rodríguez-Ortega M.J., Lenz C., Valledor L. (2015). Fourteen years of plant proteomics reflected in Proteomics: Moving from model species and 2DE-based approaches to orphan species and gel-free platforms. Proteomics.

[35] O’Farrell P.H. (1975). High resolution two-dimensional electrophoresis of proteins. J. Biol. Chem..

[36] Huang B.R., Xu C.P., Xu Y. (2008). Protein extraction for two-dimensional gel electrophoresis of proteomic profiling in turfgrass. Crop Sci..

[37] Friedman D.B., Lilley K.S., John W. (2009). Difference gel electrophoresis (DIGE). The Protein Protocols Handbook.

[38] Lilley K.S., Freidman D.B. (2004). All about DIGE: Quantification technology for differential display 2D-gel proteomics. Expert Rev. Proteom..

[39] Unlu M., Morgan M.E., Minden J.S. (1997). Difference gel electrophoresis: A single gel method for detecting changes in protein extracts. Electrophoresis.

[40] Colignon B., Raes M., Dieu M., Delaive E., Mauro S. (2013). Evaluation of three-dimensional gel electrophoresis to improve quantitative profiling of complex proteomes. Proteomics.

[41] Baerenfaller K., Grossmann J., Grobei M.A., Hull R., Hirsch-Hoffmann M., Yalovsky S., Zimmermann P., Grossniklaus U., Gruissem W., Baginsky S. (2008). Genome-scale proteomics reveals *Arabidopsis thaliana* gene models and proteome dynamics. Science.

[42] Imin N., Kerim T., Weinman J.J., Rolfe B.G. (2001). Characterisation of rice anther proteins expressed at the young microspore stage. Proteomics.

[43] Ferchab A., Capriotti A.L., Caruso G., Cavaliere C., Samperi R., Stampachiacchiere S., Laganà A. (2014). Comparative analysis of metabolic proteome variation in ascorbate-primed and unprimed wheat seeds during germination under salt stress. J. Proteom..

[44] Capriotti A.L., Borrelli G.M., Colapicchioni V., Papa R., Piovesana S., Samperi R., Stampachiacchiere S., Laganà A. (2014). Proteomic study of a tolerant genotype of durum wheat under salt-stress conditions. Anal. Bioanal. Chem..

[45] Fercha A., Capriotti A.L., Caruso G., Cavaliere C., Stampachiacchiere S., Chiozzi R.Z., Laganà A. (2016). Shotgun proteomic analysis of soybean embryonic axes during germination under salt stress. Proteomics.

[46] Capriotti A.L., Caruso G., Cavaliere C., Samperi R., Stampachiacchiere S., Chiozzi R.Z., Laganà A. (2014). Protein Profile of Mature Soybean Seeds and Prepared Soybean Milk. J. Agric. Food Chem..

[47] Aebersold R., Mann M. (2003). Mass spectrometry-based proteomics. Nature.

[48] Choi J.H., Sung Z.R. (1984). Review and Perspectives: Two-dimensional gel analysis of carrot somatic embryonic proteins. Plant Mol. Biol. Rep..

[49] Helleboid S., Hendriks T., Bauw G., Inze D., Vasseur J., Hilbert J.-L. (2000). Three major somatic embryogenesis related proteins in Cichorium identified as PR proteins. J. Exp. Bot..

[50] Gianazza E., De Ponti P., Scienza A., Villa P., Martinelli L. (1992). Monitoring by two-dimensional electrophoresis somatic embryogenesis in leaf and petiole explants from Vitis. Electrophoresis.

[51] Sallandrouze A., Faurobert M., El Maataoui M., Espagnac H. (1999). Two dimensional electrophoresis analysis of proteins associated with somatic embryogenesis development in *Cupressus sempervirens* L.. Electrophoresis.

[52] Bian F., Zheng C., Qu F., Gong X., You C. (2010). Proteomic Analysis of Somatic Embryogenesis in *Cyclamen persicum* Mill. Plant Mol. Biol. Rep..

[53] Lippert D., Zhuang J., Ralph S., Ellis D.E., Gilbert M., Olafson R., Ritland K., Ellis B., Douglas C.J., Bohlmann J. (2005). Proteome analysis of early somatic embryogenesis in *Picea glauca*. Proteomics.

[54] Flinn B.S., Roberts D.R., Newton C.H., Cyr D.R., Webster F.B., Taylor I.E.P. (1993). Storage protein gene exprcssion in zygotic and somatic embryos of interior spruce. Physiol. Plant.

[55] Lal S.K., Lee C., Sachs M.M. (1998). Differential regulation of enolase during anaerobiosis in maize. Plant Physiol..

[56] Imin N., Nizamidin M., Daniher D., Nolan K.E., Rose R.J., Rolfe B.G. (2005). Proteomic analysis of somatic embryogenesis in *Medicago truncatula*. Explant cultures grown under 6-Benzylaminopurine and 1-Napthaleacetic Acid treatment. Plant Physiol..

[57] Jong F., Mathesius U., Imin N., Rolfe B.G. (2007). A Proteome study of the proliferation of cultured *Medicago truncatula* protoplasts. Plant Proteom..

[58] Amsterdam A., Pitzer F., Baumeister W. (1993). Changes in intracellular localization of proteasomes in immortalized ovarian granulosa cells during mitosis associated with a role in cell cycle control. Proc. Natl. Acad. Sci. USA.

[59] Ito H., Iwabuchi M., Ogawa K. (2003). The sugar-metabolic enzymes aldolase and triose-phosphate isomerase are targets of glutathionylation in *Arabidopsis thaliana*: Detection using biotinylated glutathione. Plant Cell Physiol..

[60] Heringer A.S., Santa-Catarina C., Silveira V. (2018). Insights from proteomic studies into plant somatic embryogenesis. Proteomics.

[61] Agrawal G.K., Job D., Zivy M., Agrawal V.P., Bradshaw R.A., Dunn M.J., Haynes P.A., Wijk K.J.V., Kikichi S., Renaut J. (2011). Time to articulate a vision for the future of plant proteomics—A global perspective: An initiative for establishing the International Plant Proteomics Organization (INPPO). Proteomics.

[62] Subhra C., Ghasem H.S., Yang P.F., Woo S.H., Chin C.F., Chris G., Paul A.H., Mehdi M., Komatsu S. (2015). Proteomics of Important Food crops in the Asia Oceania Region: Current Status and Future Perspectives. J. Proteome Res..

[63] Campos N.A., Paiva L.V., Panis B., Carpentier S.C. (2016). The proteome profile of embryogenic cell suspensions of *Coffea arabica* L.. Proteomics.

[64] Correia S., Vinhas R., Manadas B., Lourenço A.S., Veríssimo P., Canhoto J.M. (2012). Comparative proteomic analysis of auxin-induced embryogenic and nonembryogenic tissues of the Solanaceous tree *Cyphomandra betacea* (Tamarillo). J. Proteome Res..

[65] Tan H.S., Liddell S., Abdullah M.O., Wong W.C., Chin C.F. (2016). Differential proteomic analysis of embryogenic lines in oil palm (*Elaeis guineensis* Jacq). J. Proteom..

[66] De Carvalho Silva R., Travassos Carmo L.S., Gomes Luis Z., Silva L.P., Scherwinski-Pereira J.E., Mehta A. (2014). Proteomic identification of differentially expressed proteins during the acquisition of somatic embryogenesis in oil palm (*Elaeis guineensis* Jacq.). J. Proteom..

[67] Baba A., Nogueira F., Pinheiro C., Brasil J., Jereissati E., Juca T., Soares A., Santos M., Domont G., Campos F. (2008). Proteome analysis of secondary somatic embryogenesis in cassava (*Manihot esculenta*). Plant Sci..

[68] Kumaravel M., Uma S., Backiyarani S., Saraswathi M.S., Vaganan M.M., Muthusamy M., Sajith K.P. (2017). Differential proteome analysis during early somatic embryogenesis in Musa spp. AAA cv. Grand Naine. Plant Cell Rep..

[69] Guzmán-García E., Sánchez-Romero C., Panis B., Carpentier S.C. (2013). The use of 2D-DIGE to understand the regeneration of somatic embryos in avocado. Proteomics.

[70] Sghaier-Hammami B., Driraa N., Jorrín-Novob J.V. (2009). Comparative 2-DE proteomic analysis of date palm (*Phoenix dactylifera* L.) somatic and zygotic embryos. J. Proteom..

[71] Heringer A.S., Reis R.S., Passamani L.Z., de Souza-Filho G.A., Santa-Catarina C., Silveira V. (2017). Comparative proteomics analysis of the effect of combined red and blue lights on sugarcane somatic embryogenesis. Acta Physiol. Plant..

[72] Niemenak N., Kaiser E., Maximova S.N., Laremore T., Guiltinan M.J. (2015). Proteome analysis during pod, zygotic and somatic embryo maturation of *Theobroma cacao*. J. Plant Physiol..

[73] Noaha A.M., Niemenaka N., Sunderhausb S., Haaseb C., Omokoloa D.N., Winkelmannc T., Braun H.P. (2013). Comparative proteomic analysis of early somatic and zygotic embryogenesis in *Theobroma cacao* L.. J. Porteom..

[74] Tan B.C., Chin C.F., Liddell S., Alderson P. (2013). Proteomic analysis of callus development in *Vanilla planifolia* Andrews. Plant Mol. Biol. Rep..

[75] Cassells A.C., Curry R.F. (2001). Oxidative stress and physiological, epigenetic and genetic variability in plant tissue culture, implications for micropropagators and genetic engineers. Plant Cell Tissue Organ Cult..

